# Discretionary foods have notable environmental and expenditure relevance across meat and plant protein preferences

**DOI:** 10.1038/s41538-026-00721-x

**Published:** 2026-01-20

**Authors:** Jelena Meinilä, Rachel Mazac, Henna Vepsäläinen, Juha-Matti Katajajuuri, Hanna L. Tuomisto, Mikael Fogelholm, Maijaliisa Erkkola, Jaakko Nevalainen

**Affiliations:** 1 Department of Food and Nutrition, University of Helsinki, Helsinki, Finland; 2 Department of Agricultural Sciences, University of Helsinki, Helsinki, Finland; 3 Stockholm Resilience Center, Stockholm University, Stockholm, Sweden; 4 Natural Resources Institute Finland, Helsinki, Finland; 5 Helsinki Institute of Sustainability Science, University of Helsinki, Helsinki, Finland; 6 Faculty of Social Sciences, Unit of Health Sciences, Tampere University, Tampere, Finland

**Keywords:** Risk factors, Environmental impact

## Abstract

Real-world data help clarify the contribution of food to nutrition, the environment, and food expenditure. We studied the implications of a hypothetical transition in protein sources for these sustainability dimensions using loyalty-card holders’ (*n* = 22,901) food purchases. Six consumer clusters were identified via sequence analysis, representing realistic transitions in protein sources alongside other food consumption changes. Cross-sectional comparisons revealed that higher expenditure of Plant-based and Fish clusters per 2500 kcal was largely driven by other food groups than the protein sources, while the protein source expenditure was relatively consistent across clusters. Environmental impact differences were largely attributable to the protein sources, with meat and fish contributing the most. Aside from protein sources, discretionary foods accounted for 22% of spending and contributed up to 17–32% of environmental impacts. Therefore, alongside protein source changes, reducing discretionary food consumption could yield notable environmental benefits and allow reallocation of expenditure towards more nutritious foods.

## Introduction

Food systems are responsible for about a third of all greenhouse gas emissions (GHGEs, primarily carbon dioxide, methane, and nitrous oxide)^1^, and agriculture is the main contributor to land and water use, biodiversity loss, and use of nitrogen and phosphorus^2^. On average, the production of animal-based foods has larger environmental impacts than that of plant-based foods^3^. Therefore, there is a growing global need to shift from animal-based diets to more plant-based diets^4^.

Animal-based foods (meat, dairy, fish, egg) have a notable role as a protein source, as in high-income countries in North America and Europe, they provide 60–70% of protein intake^5,6^. Thus, protein sources are central in the transition towards more ecologically sustainable diets, although environmental impact assessment beyond GHGE is still necessary.

Protein source foods also provide numerous nutrients besides protein, such as iron and saturated fatty acids (SFAs) from meat, vitamin B_12_ from meat and dairy, vitamin D from fish, and folate and fiber from legumes (https://fineli.fi/fineli/en/index). Some of the essential nutrients lost through a shift from one protein source to another can be compensated for by other food groups, whereas vitamin B_12_ is only naturally available from animal-based foods. Therefore, estimation of the overall implications for nutrition of the total food consumption pattern is essential when analyzing the nutritional consequences of food transition towards a more plant-based diet. Protein sources also vary in cost, influencing their accessibility and affordability.

Several studies have been conducted in which considerations regarding cultural acceptability are limited or based on subjective decisions on how much the modeled diet is allowed to differ from the current diet^7–10^. Previous work in this area has suggested that transitioning towards more sustainable protein sources is a gradual process and more likely to occur between similar protein sources: from red meat to poultry, from poultry to fish, and from fish to plant-based protein sources^11^. In earlier analyses, we have also identified six clusters based on the main protein sources of the purchases^11^. We believe that these real, self-selected consumption patterns are more appealing to adjacent consumer groups than hypothetical patterns based primarily on assumptions about sustainability or artificial acceptability criteria. The real-life clusters also allow realistic comparison of impacts of “discretionary foods”, i.e., foods not essential to meet nutrient requirements and often including high content of energy, saturated fat, sugar, salt, or alcohol^12^. Previous research suggests that this food group may hugely contribute to the environmental impacts of food consumption in Western societies^13–15^. Comparison of several environmental impacts across different consumption patterns is, however, missing in research to date.

We built upon and used the six previously identified clusters based on selected protein sources^11^ as cross-sectional data to mimic a longitudinal, stepwise transition towards more plant-based diets. We examined: (1) would a hypothetical transition from one cluster to another cluster affect food expenditure?, (2) what are the environmental impacts of total food purchases and specific food groups?, and (3) how would the hypothetical transition affect the nutritional composition of the food purchases?

We use real, empirical data to illustrate a potential, step-by-step transition from red meat-based to plant-based protein sources. However, because the clusters comprise separate households, differences between clusters may extend beyond protein sources alone. Therefore, while we interpret the observed clusters as representing potential transition pathways towards plant-based food consumption, whether a household’s entire purchase profile would change when transitioning between protein sources remains unknown.

## Results

### Characteristics of participants by cluster

The members of the Plant-based cluster were more often women than were the members of the other clusters (Table 1). The members of the Fish cluster had a Master’s degree or higher more often than the other clusters. The members of the Plant-based cluster were the youngest (mean 38 years), and those of the Fish cluster were the oldest (56 years). The members of the Plant-based cluster had more often a monthly scaled household income of <1000 €, and the Fish cluster had more often a monthly scaled household income of 4000 € or more compared with the others.

**Table 1:** Characteristics of 22,901 loyalty-card holders by cluster

	Red meat (*N* = 6554)	Red meat mixed (*N* = 9580)	Red meat & Poultry (*N* = 1980)	Mixed (*N* = 3265)	Fish (*N* = 464)	Plant-based (*N* = 1058)
Sex, *n* (%)						
Men	2487 (38)	3364 (35)	576 (29)	948 (29)	161 (35)	227 (22)
Women	4067 (62)	6216 (65)	1404 (71)	2317 (71)	303 (65)	831 (79)
Age, mean (SD)	51 (14)	48 (15)	43 (15)	47 (17)	56 (15)	38 (13)
Highest education, *n* (%)						
Primary school or lower	668 (10)	584 (6)	61 (3)	136 (4)	14 (3)	22 (2)
Upper secondary school	3026 (46)	3611 (38)	639 (32)	951 (29)	102 (22)	289 (27)
Bachelor’s degree or equivalent	1908 (29)	3175 (33)	732 (37)	1031 (32)	141 (30)	358 (34)
Master’s degree or higher	941 (14)	2183 (23)	546 (28)	1138 (35)	205 (44)	388 (37)
Other or missing	11 (0.2)	27 (0.2)	20 (0.1)	9 (0.3)	2 (0.4)	1 (0.1)
Loyalty, *n* (%)						
61–80%	2099 (32)	3994 (42)	757 (38)	1489 (46)	189 (41)	470 (44)
81–100%	4455 (68)	5586 (58)	1223 (62)	1776 (54)	275 (59)	588 (56)
Scaled household income (€/month)						
<1000	430 (7)	759 (8)	191 (10)	349 (11)	34 (7)	209 (20)
1000–1999	1250 (19)	1379 (14)	246 (12)	363 (11)	31 (7)	133 (13)
2000–2999	2013 (31)	2751 (29)	590 (30)	866 (27)	125 (27)	295 (28)
3000–3999	1521 (23)	2206 (23)	437 (22)	692 (21)	99 (21)	202 (19)
4000 or more	919 (14)	1881 (20)	385 (19)	768 (24)	131 (28)	165 (16)
Missing	421 (6)	604 (6)	131 (7)	227 (7)	44 (10)	54 (5)

### Purchase volumes of food per 2500 kcal by cluster

**Fig. 1:**
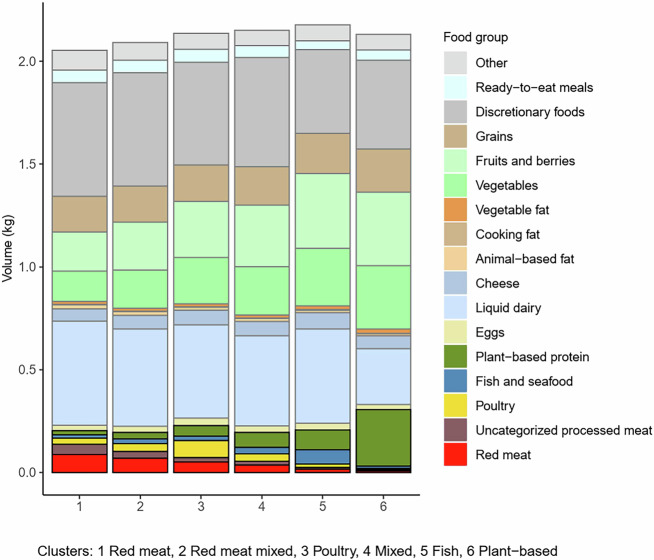
Food group-specific mean purchase volumes (kg) per 2500 kcal of purchases among 22,901 loyalty-card holders.

As the clusters were identified based on the main protein source of the purchases, it is logical that they differed largely in purchase volumes of the protein sources (Fig. 1). In addition to the protein sources, the most important differences among the clusters were attributable to fruit, vegetable, and liquid dairy purchases. Fruit and vegetable purchases increased consistently, while red and processed meat purchases decreased from one cluster to another (∆% (Plant-based, Red meat): fruits and berries +89%, vegetables +107%, red meat −89%, Supplementary Data 1). The members of the Plant-based cluster bought considerably less liquid dairy products than the others, in absolute terms (Fig. 1) and in proportion to the total purchases (13% vs. 20–25%, Supplementary Data 1). Discretionary foods’ purchase volume was slightly smaller among members of the Fish and Plant-based clusters in absolute terms (Fig. 1) and in proportion to total purchases (Fish 19%, Plant-based 20%, others 23–27% of total purchases, Supplementary Data 1).

### Expenditure on food per 2500 kcal by cluster

**Fig. 2:**
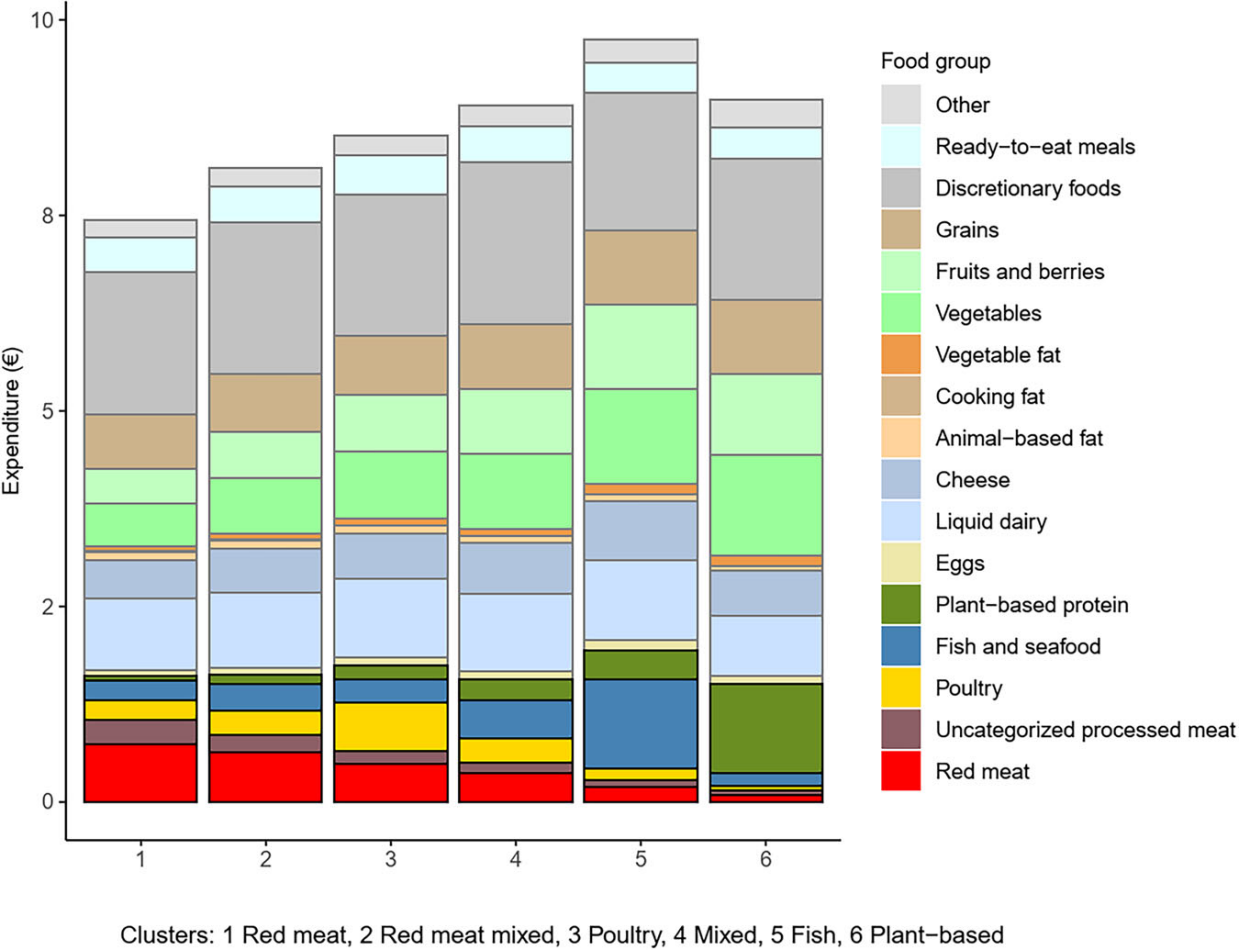
Food group-specific mean expenditure per 2500 kcal of purchases among 22,901 loyalty-card holders.

Members of the Fish cluster spent the most money on food (mean 9.8€), followed by the Plant-based (9.0€) and Mixed clusters (8.1€). Members of the Red meat cluster spent the least money on food (7.4€) (Table 2 and Fig. 2). Differences among the clusters in food expenditure were not attributable to the main protein source (Fig. 2). For example, members of the plant-based and red meat clusters spent approximately the same amount of money on the main protein sources (although protein content in the purchases was smaller, as seen later in the section Nutrient content of foods per 2500 kcal by cluster). The main contributors to the differences were expenditures on fruits and vegetables (in fruits and berries, maximum ∆% (Fish, Red meat): +143%, in vegetables, maximum ∆% (Plant-based, Red meat): +139%) (Supplementary Data 1).

**Table 2:** Expenditure, nutrient contents, and environmental impacts of total food purchases per 2500 kcal among 22,901 loyalty-card holders by cluster

	Red meat (*N* = 6554)	Red meat mixed (*N* = 9580)	Red meat & Poultry (*N* = 1980)	Mixed (*N* = 3265)	Fish (*N* = 464)	Plant-based (*N* = 1058)
	Mean (SD)	Mean (SD)	Mean (SD)	Mean (SD)	Mean (SD)	Mean (SD)
*Expenditure per 2500 kcal, €*	7.4 (1.9)	8.1 (2.1)	8.5 (2.1)	8.1 (2.4)	9.8 (2.8)	9.0 (2.4)
*Nutrient content per 2500 kcal*						
Protein, g	97 (14)	97 (16)	104 (19)	94 (18)	97 (19)	83 (17)
Sucrose, g	67 (23)	68 (24)	65 (23)	69 (28)	64 (24)	69 (22)
Fiber, g	20 (5)	22 (6)	24 (7)	26 (8)	30 (9)	34 (10)
SFA, g	45 (8)	44 (9)	42 (9)	42 (10)	40 (10)	36 (10)
Vitamin B12, µg	6.6 (2.4)	6.6 (2.6)	6.5 (2.3)	6.5 (3.3)	8.0 (3.5)	3.7 (2.1)
Vitamin D, µg	10.1 (3.6)	9.4 (3.7)	8.7 (3.6)	9.0 (4.3)	11.7 (5.3)	6.3 (3.3)
Folate, mg	244 (59)	265 (83)	287 (88)	296 (94)	326 (85)	347 (99)
Calcium, mg	1330 (346)	1330 (347)	1310 (349)	1350 (393)	1460 (419)	1230 (451)
Iron, mg	11.4 (2.22)	11.9 (2.34)	12.6 (2.59)	12.8 (2.91)	13.6 (2.85)	15.0 (3.37)
Salt, g	7.2 (1.3)	6.8 (1.2)	6.5 (1.2)	6.3 (1.4)	6.3 (1.5)	5.8 (1.3)
*Environmental impact per 2500* *kcal*						
GHGE, kg CO_2_-eq.	4.9 (0.9)	4.9 (1.0)	5.0 (1.0)	4.5 (1.0)	4.1 (0.9)	3.6 (0.9)
Marine eutrophication, g N-eq.	6.1 (1.0)	5.9 (0.9)	6.0 (1.0)	5.5 (1.0)	5.3 (1.1)	4.7 (1.0)
Freshwater eutrophication, g P-eq.	2.0 (3.8)	2.0 (0.4)	2.0 (0.4)	2.0 (0.6)	2.5 (0.9)	1.5 (0.4)
Land use, m^2^a crop-eq.	6.0 (1.0)	5.6 (0.9)	5.4 (1.0)	4.8 (1.0)	4.3 (0.8)	3.6 (0.8)
Water use, m^3^	0.23 (0.05)	0.24 (0.06)	0.26 (0.06)	0.26 (0.08)	0.30 (0.10)	0.24 (0.07)

*GHGE* greenhouse gas emission, *N-eq.* nitrogen equivalent, *P-eq.* phosphorus equivalent, *crop-eq.* arable cropland equivalent.

A large proportion of the total food expenditure was attributable to discretionary foods in all clusters (from 18% in Plant-based to 24% in Red meat) (Supplementary Data 1 and Fig. 2). In all clusters, the main food groups among discretionary foods in terms of expenditure were alcoholic and non-alcoholic beverages, sweet bakery products, and sweets (Supplementary Fig. 1).

### Environmental impacts of foods per 2500 kcal by cluster

**Fig. 3:**
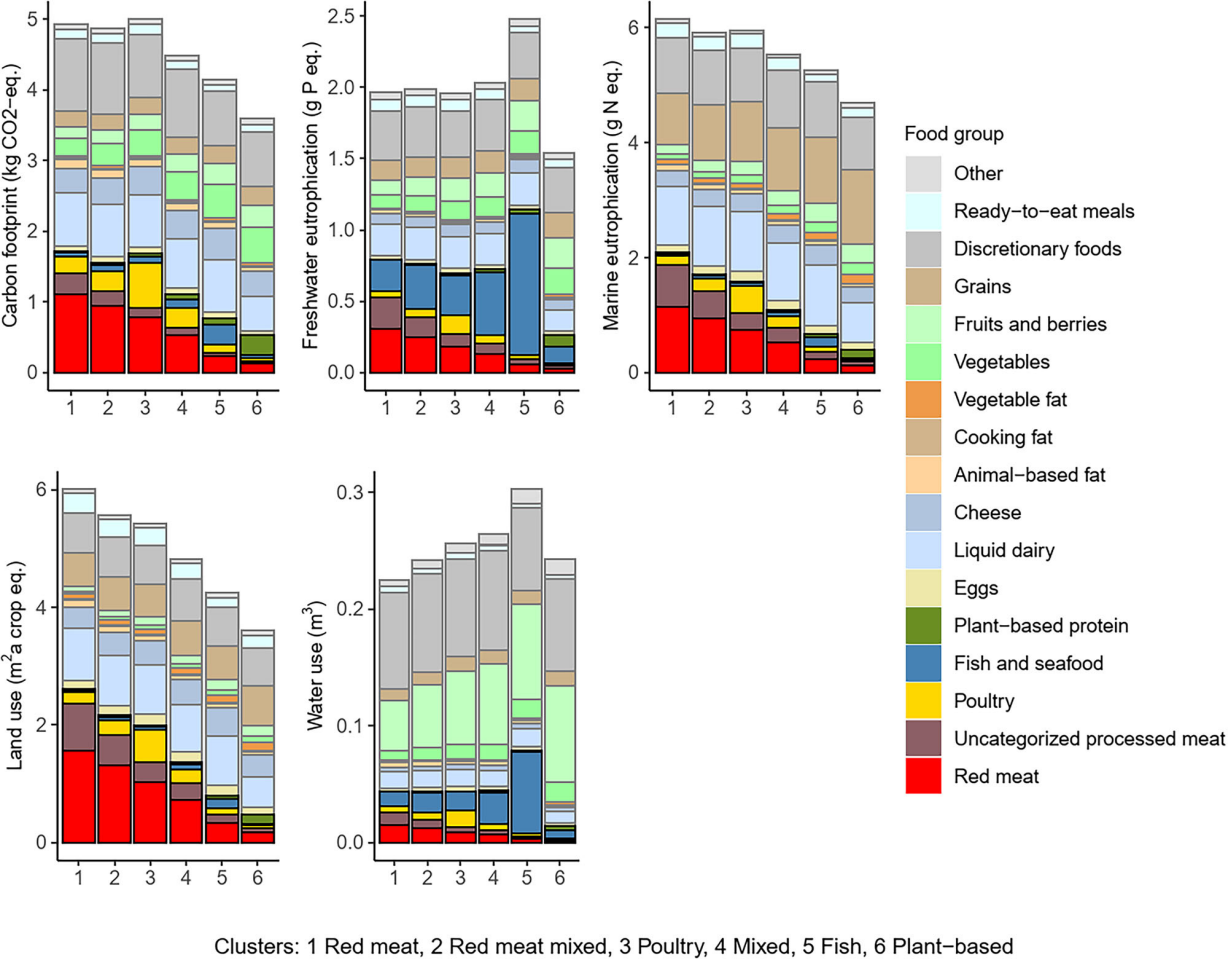
Food group-specific greenhouse gas emissions, freshwater eutrophication, marine eutrophication, land use, and consumptive water use per 2500 kcal of purchases among 22,901 loyalty-card holders.

For GHGEs, land use, and marine eutrophication, the impacts of the purchases decreased from meat-dominant (Red meat, Red meat mixed, Red & poultry) to Plant-based clusters (Table 2 and Fig. 3). GHGE of total purchases was similar in the purchases of Red meat, Red meat mixed, and Red meat & poultry clusters, after which the emissions diminished stepwise through Fish to the Plant-based clusters (maximum ∆% (Plant-based, Red meat & poultry): −28%). Although several food groups contributed to the GHGE, land use, and marine eutrophication, the major contributors to the differences among the clusters were the main protein sources (Fig. 3).

Differences in freshwater eutrophication among the clusters were mainly explained by fish content, being the highest in the purchases of the Fish cluster (2.5 g P eq., from which 40% originated from fish, Supplementary Data 1), and the lowest in the Plant-based cluster (1.5 g P eq., from which 20% originated from fish) (Fig. 3). A large portion of the freshwater eutrophication impact of fish came from fish that could not be classified as either wild-caught or farmed due to lack of information on the species (34-85% from total impact of fish, depending on the cluster) (Supplementary Table 1**)**. Consumptive water use and content of fruits and fish tended to be associated across the clusters. Water use was the highest for the Fish cluster and the lowest for the Red meat cluster (Table 2 and Fig. 3).

Discretionary foods had a large contribution to all environmental impacts in all clusters (on average 17–32% of total purchases, depending on the environmental impact category) (Fig. 3, Supplementary Data 1). For GHGE, the largest contributors within the discretionary foods were beverages, including both alcoholic and non-alcoholic drinks (Supplementary Fig. 2).

### Nutrient content of foods per 2500 kcal by cluster

**Fig. 4:**
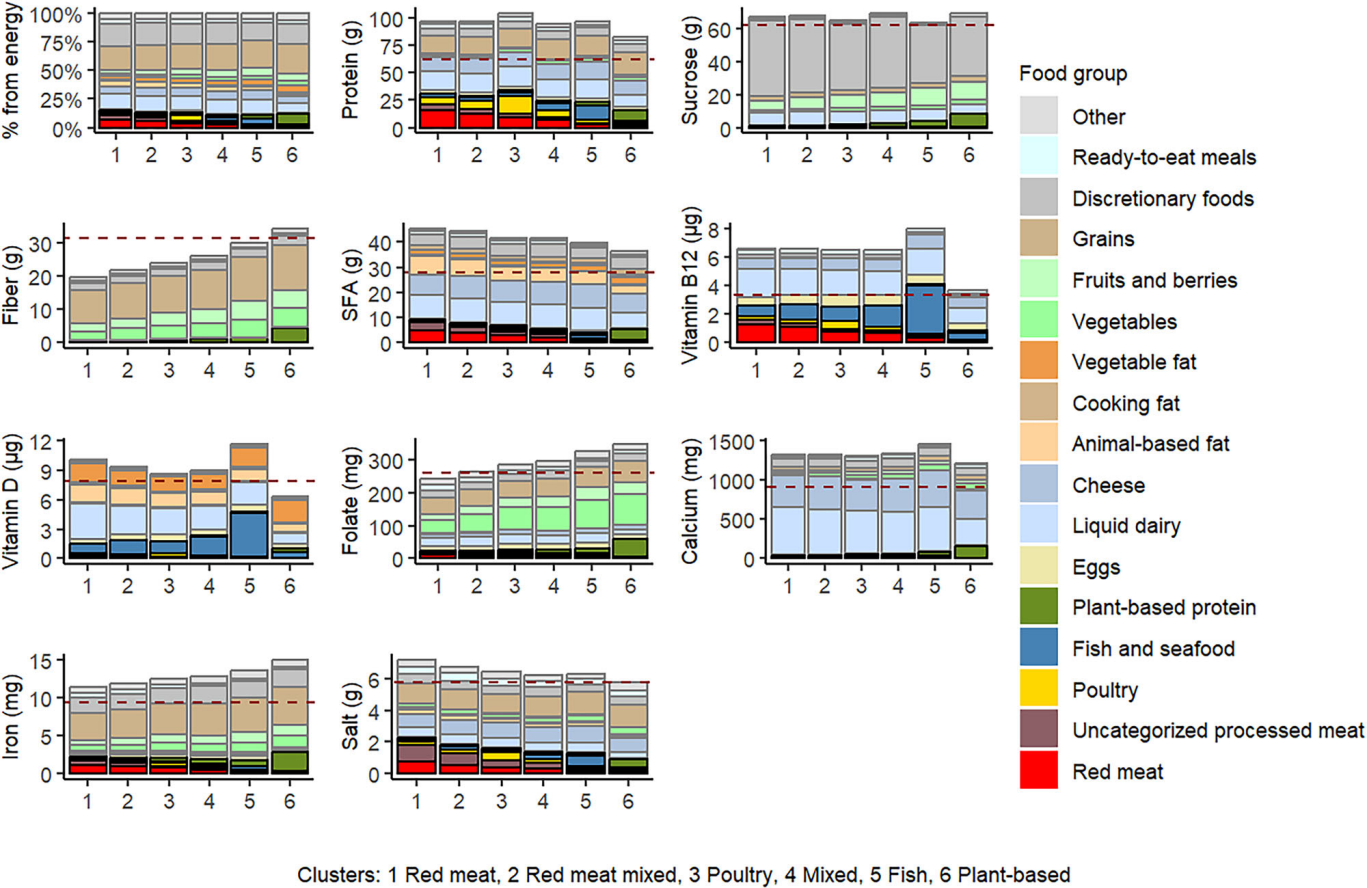
Food group-specific nutrient content per 2500 kcal of purchases among 22,901 loyalty-card holders. The dashed horizontal lines represent recommended intake (protein (10–20E%), added/free sugar for sucrose ( < 10E%), SFA ( < 10E%), and fiber ( ≥ 3 g/MJ)), average requirement (vitamin D, folate, calcium, iron), provisional average requirement (vitamin B12), or upper limit for chronic disease prevention (salt) scaled to energy intake of 2500 kcal (10.5 MJ). The reference values are originally defined for 10 MJ (2388 kcal) in the Nordic Nutrition Recommendations 2023^25^.

Folate, fiber, and iron contents of the total purchases increased stepwise as red meat content of the cluster decreased (Table 2 and Fig. 4). These differences in nutrient content were mainly attributable to differences in fruit, vegetable, and grain purchases (Fig. 4), with members of the Plant-based and Fish clusters purchasing fruits, vegetables, and high-fiber grains the most.

The opposite was observed for salt and SFA contents, which decreased stepwise as red meat purchases in the cluster decreased. Although in all clusters most of the salt was derived from food groups other than the main protein source, the differences between the clusters were mainly related to the main protein sources of the purchases. Vitamins B_12_ and vitamin D content were the lowest in the purchases of members of the Plant-based cluster and the highest in the Fish cluster. Protein content was lower in the Plant-based cluster than in the other clusters.

Discretionary foods were responsible for a large amount of energy (average 18% of total purchases) in all clusters (Supplementary Data 1 and Fig. 4) and contributed markedly to sucrose contents in all clusters (Fig. 4, Supplementary Fig. 3). Among discretionary foods, the sucrose content of purchases was the highest in the Red meat cluster.

## Discussion

A notable finding was that expenditure for the protein sources was similar across the clusters, regardless of the choice of the main protein source. The mean protein supply was lower in the Plant-based cluster than in the others, but was well within the recommended intake range (10–20% of total energy supply, which is 63–125 g in 2500 kcal/d intake)^16^. Another main finding was that a hypothetical step from the Red meat cluster to the Poultry cluster would bring little, if any, environmental benefit, whether based on the protein sources alone or the entire purchases. In contrast, better environmental and nutritional gains would be achieved by transitioning from any meat-dominant cluster to a Plant-based cluster. Notably, discretionary foods played a large role across all clusters, comprising a substantial proportion of food expenditure, while having significant negative impacts on both environmental footprints and nutrient content of food purchases.

Because of minimal differences in expenditure (per energy unit) in protein source purchases between the clusters, we infer that a shift from meat to fish or plant-based protein sources does not seem to be an economic obstacle for the average Finnish household. Other studies have also shown that plant-based protein sources, such as legumes, have been, on average, cheaper than or priced equal to meat^17,18^. In a previous study, plant-based protein products were bought, on average, at a lower price than meat products^11^. However, it is possible that the perception of price is more important than the actual price in food choice preferences^19^. In previous studies, both meat-eaters and those consuming plant-based foods or transitioning to plant-based food consumption have considered the price motive to be important^20–23^. Motives also seem to vary depending on the food group in question.

Independent of the cluster, the amount of money spent on discretionary foods was large, at least one-fifth of the total food expenditure. Food serves purposes beyond nutritional value, such as regulating feelings, social influences, and constructing cultural identity^24^, which complicates the idea of substituting food choices across different food groups, e.g., substituting fruits and vegetables for discretionary foods.

Unexpectedly, those choosing more unprocessed poultry and less red and processed meat (including both processed red meat and processed poultry) did not have smaller GHGEs from protein sources or from total purchases than those who bought mainly red and processed meat. This may have been because a large part of the “replaced” red and processed meat (by poultry) was processed red meat, which may contain ingredients with low GHGE per kg, e.g., potato extracts in many sausages, reducing the overall GHGE of the red meat product. These findings support the meat recommendation of the food-based dietary guidelines of the Nordic Nutrition Recommendations 2023, which emphasize not replacing red meat with poultry, instead replacing it with plant-based protein sources and sustainable fish products^25^, and a recent Finnish study suggesting poultry intake as one of the primary foods to contribute to global biodiversity loss^26^.

Although many of the foods included in discretionary foods have relatively low environmental impact per kg of product compared with, for example, meats, the high consumption volume makes them a significant food group. The finding is noteworthy, as many discretionary foods have small environmental impacts per mass unit, which may take attention away from their overall impact as part of the whole diet.

In previous Nordic studies, discretionary foods have played a relatively large role in environmental impacts^14,15,27^. Given that discretionary foods are non-essential and often unhealthy, they represent an avoidable burden to the environment. Thus, the results suggest a potential reduction of environmental impacts through reducing consumption of discretionary foods in all population groups.

The relatively large contribution of fruits and vegetables to consumptive water use has also been reported earlier, e.g., in a Swedish study^28^. However, the measure of consumptive water use does not directly inform about water overconsumption because it does not consider water scarcity in the production area. Currently, a lack of data hinders research on water scarcity impacts of Finnish food consumption^29^. In a US study, large scarcity-weighted water footprints were found for meat, especially beef, and some fruits, nuts, and seeds^30^. The study is, however, not fully comparable with Finnish food consumption because of the different agricultural conditions of some foods. In Finland, which is highly self-sufficient in beef production, most of the agricultural production is rainfed^31^. Therefore, consumptive water use for beef production in Finland is minimal, and water scarcity is generally low^32^. In contrast, certain imported foods, such as some fruits, rely on irrigation, notably contributing to consumptive water use^33^. Some of the fruits may come from water-scarce areas.

The purchases of the members of the Fish and Plant-based clusters had overall the most favorable nutrient contents, in accordance with earlier results^9,10^. Although we did not consider dairy products as one of the main protein sources in our analysis, they are an important food group in the sustainability transition because of their significant role in food culture, in environmental impact, and as a source of many nutrients. In this study, a trade-off of lower environmental impacts of the purchases in the Plant-based cluster was the relatively low vitamin D intake because of lower dairy content than in the other clusters. According to our results, Finland’s otherwise successful fortification program of dairy products and fat spreads^34^ might not be sufficient to cover the vitamin D intake of the members of the Plant-based cluster. The Finnish Nutrition Recommendations^16^ recommend vitamin D supplementation for individuals who do not regularly consume fish or products fortified with vitamin D.

There are feasibility issues to be resolved in a household’s transition towards healthier and sustainable food consumption. Firstly, protein source transition seems to be very challenging, despite the purchase of protein not being an affordability issue in most households. The persistence of protein source preferences was shown in a previous paper, where most people adhered to their choice of protein (mainly meat) from week to week^11^. Secondly, although a shift from red meat to poultry seems to bring little environmental benefit, this particular shift seems to be more acceptable to consumers than shifting directly to fish or plant-based protein source consumption^11^. Hence, skipping the step from meat to poultry might not be realistic in the current context. Thirdly, although reducing discretionary food purchases would be beneficial in terms of nutrition, environmental impacts, and expenditure, like other food-related changes, this may be challenging. As with meat, some (if not all) of the discretionary foods, such as coffee and alcoholic beverages, have a strong position in the Finnish food culture^35–37^. Many are highly palatable^38,39^ and aggressively marketed^40^, and, according to our results, apparently well-accepted and valued across the population.

Given the urgent need for change, reforming the sociocultural environment towards more acceptable sustainable food choices, namely more sustainable fish and plant-based foods and less animal-sourced and discretionary foods, is essential. Such reforms call for prioritizing systemic transformations, such as incentives for cultivating legumes for human consumption to increase availability, restructuring current policy incentives (e.g., taxes or agricultural incentives), and implementing restrictions on the marketing of unhealthy discretionary foods. The food industry and retailers, who act as intermediaries between suppliers and consumers, have untapped potential to promote healthier and more sustainable production and consumption patterns^41^. Furthermore, policy decisions must address the affordability issues associated with certain sustainable fish products to ensure broader access and adoption of these options. However, for any large-scale change to be truly effective, it must broadly consider the sociocultural needs of the population it affects^42^.

A unique strength of this study was the use of food purchase data with an exceptionally large dataset in the context of nutrition research and a larger sample size and longer timespan than could be collected using traditional methods. Loyalty-card data are less reliant on self-reporting than traditional dietary intake data. The self-selected data brought realism to the analysis in terms of cultural acceptability, which is difficult to evaluate in studies based on theoretical and modeled diets. Another strength is the data on actual individual-level food expenditure, which in food consumption research is usually self-reported or approximations based on statistics. Furthermore, in the LCA, particular emphasis was given to differences between domestic and imported foods, enhancing the validity of the data.

We were unable to calculate absolute daily purchases of individuals because the cardholder usually buys food for the whole household. In addition, not all of the household’s food is purchased from the retailer in question. Moreover, the household members’ diets may also differ from each other. However, we have shown that when comparing the food purchases of the cardholders with food intake data derived from food frequency questionnaires collected for the same individuals, the relative validity for most food groups is acceptable^43^. Unlike many previous studies, we did not include processed meat in discretionary foods^13,44,45^. Although the impacts of processed meat are separately visible in our results, this difference in classification may cause confusion for some readers. Differences in how discretionary foods are defined should be considered, especially when comparing the environmental impacts and salt content of discretionary foods across studies. In our study, these impacts were significant for processed meat but were not included in the discretionary food implications. Additionally, coffee has rarely been considered part of discretionary foods in previous research, but in our study, it contributed notably to marine eutrophication and land use within this category. In our view, classifying coffee as a discretionary food emphasizes its contribution to avoidable environmental impacts associated with discretionary consumption.

The validity of the magnitudes of eutrophication from fish is affected by the fact that species and production method for some of the products were not identified and therefore were classified as “uncategorized fish products”. The environmental impact coefficients for “uncategorized fish products,” which accounted for a significant share of the freshwater eutrophication impact, are therefore more uncertain than those of other captured and farmed fish. The large contribution of fish to freshwater eutrophication was, however, similar to another study of Finnish diets^46^. Another consideration is that captured fish from Finnish lakes and the Baltic Sea remove nitrogen and phosphorus from the water system, thereby reducing eutrophication^47^, which was not considered in the impacts. In addition, the French environmental impact data probably did not rigorously reflect the environmental impacts of Finnish food consumption. However, the directions and patterns of at least the most established environmental impacts of foods in different studies across several countries point in the same direction^4,8,14,46^. The environmental impacts reported in this study should not be considered as absolute impacts but as indications of relative differences between the clusters and food groups.

Although our idea in the study was that the purchase profiles of the protein preference clusters could indicate a realistic transition pathway from meat-dominant to more sustainable food purchase profiles, whether the whole purchase profile would change following an individual’s shift from one protein source to another remains unknown.

The hypothetical protein source transition path that was derived from a large cross-sectional self-selected purchase data showed stepwise improvements in fiber, SFA, folate, iron, and salt content and in some environmental impacts, including GHGE, when moving towards plant-based protein source purchases. The step from red meat to partial replacement with poultry would bring little environmental benefit; better nutritional gains were achieved by moving from red meat dominance to fish or plant-based dominant purchases. Although the need to reduce discretionary food consumption for health reasons is widely recognized, the environmental impact of these foods also merits increased attention; a notable potential for reducing environmental impact lies in the reduction of their purchases across all population groups.

According to our results and those of others, a sustainability transition, including a transition towards more plant-based protein sources and a reduction of discretionary foods, is mainly not an affordability issue in high-income countries, apart from some expensive sustainable fish products. Instead, the obstacles for the transition of individuals/households lie among other determinants of behavior. Additionally, it is crucial not to place the burden of change solely on individual consumers but to prioritize systemic transformations, including shifts towards more sustainable agricultural practices, value chain adjustments, improved food distribution systems, and policy incentives that make healthy and sustainable choices more accessible and affordable for everyone. The Nordic Nutrition Recommendations, in line with the results of this study, provide a crucial framework for policymakers and other stakeholders towards a healthier and more sustainable food system.

## Methods

### Purchase data and participants

Data originates from the loyalty-card holders of the largest food retail chain in Finland, namely the S Group^48^. The retailer held a market share of 46% of the Finnish food retail sector in 2018 when data were collected. During the data collection period, 2.4 million households in Finland possessed the S Group’s customer loyalty card, constituting 88% of all households in the country. All individuals aged 18 years or older with a loyalty card and an email address in the retailer’s database were contacted via email to consent to the release of their purchase data for the research. Consenting respondents were also invited to complete an electronic questionnaire, collecting additional background information such as household income, highest educational attainment, and self-reported loyalty (i.e., how much (%) of the food purchases the participant buys from the S Group retailer).

Of the 47,066 consenting loyalty-card holders, we included those who completed the background questionnaire (*n* = 36,621). From the questionnaire, we derived how much and what proportion of their total food purchases the participants bought from the retailer. We selected those who made at least 50 kg of purchases during the year and who reported buying 61% or more of their food purchases from the retailer. We have previously shown that the purchases associated more strongly with the respondent’s self-reported food intake frequency (measured using a food frequency questionnaire) among the most loyal customers (loyalty >60%)^43^. Based on the above exclusions, the final number of participants was 22,901. All food purchases from the year 2018 were included in the data (nutritional supplements, such as vitamin and mineral supplements, were excluded). In this study, we used the 12-month purchase data aggregated to annual purchase volume (kg) and expenditure (€) per 2500 kcal (10.5 MJ) of purchased energy to represent the population's average daily energy requirement^25^.

Each participant provided electronic consent for the collection and use of their purchase data and questionnaire data for research. The study protocol was approved by the University of Helsinki Review Board in Humanities and Social and Behavioral Sciences (Statement 21/2018).

### Background characteristics of participants

The participant’s highest educational attainment and the household’s monthly income were collected by questionnaire in four categories from primary school or below to Master’s degree or higher. Participants reported their household’s monthly income by selecting one of seven predefined categories. The average value for each income category was divided by the square root of the household size to calculate the adjusted monthly household income (Organisation for Economic Co-operation and Development (OECD) square root scale). The resulting income was categorized into five groups. The age and sex of the primary cardholder were derived from the retailer’s database.

### GHGEs assessment

The methodology for evaluating GHGEs has been extensively described elsewhere^49^. To summarize, 3435 product groups were ascribed a GHGE coefficient (measured in kilograms of CO_2_-equivalent) with the functional unit of 1 kg of retail-purchased food. Indicator products were selected to represent these product groups, with one indicator product usually standing in for multiple product groups. Approximately 100 different indicator products were chosen based on available and suitable Life Cycle Assessment (LCA) studies in the Finnish retail context^49^. The GHGE coefficient of an indicator product was computed as the weighted average of the GHGEs of the most sold foods within the corresponding product group.

The primary life cycle phases considered in the assessment encompassed the production of inputs for agriculture, agricultural primary production, food processing, packaging, storage (pre-retail), and transportation, with exclusions of food waste, land use changes, and alterations in soil carbon stocks due to insufficient data. Data on storage, packaging, transportation, and their GHGEs were derived from Finnish and international databases^49^.

The purchase volume (in kilograms) of each product group of each loyalty card holder was multiplied by the corresponding indicator product’s GHGE coefficient to calculate customers’ product group-specific and total purchase GHGE.

### Land use, consumptive water use, and freshwater and marine water eutrophication assessment

We utilized LCA data for specific food products, aligned with ingredient-level food groups, to assess eutrophication, water use, and land use impacts (expressed per kilogram of food product). This information was sourced from the Agri-footprint database (Blonk Consultants) and the Agribalyse 3.0 database (French Agency for Ecological Transition, 2020) and analyzed using OpenLCA 1.10.3 software (GreenDelta, 2007).

Following previously published methods for assessing impacts of self-selected diets in Finland^46^, Agribalyse, a comprehensive French Life Cycle Inventory (LCI) analysis database featuring information on over 2500 products produced in France, was employed for its multi-indicator nature^50^. To adapt to products with a significant import-to-export ratio (>1), indicating substantial importation into Finland, product inventory data originally based on French average electricity use were modified to reflect electricity use in Europe, excluding Switzerland, as per FAOSTAT data by the Food and Agriculture Organization of the United Nations. Conversely, products with an import ratio <1, such as livestock products and grains for livestock feed, were considered ‘produced in Finland,’ with their inventory data adjusted to Finnish average electricity use. Information on livestock feed cultivated in Finland was derived from Finnish grains included in the Agri-footprint life cycle inventory database. We double-checked the selected products from the Agribalyse database with other databases that have previously been collated for Finnish food systems. Food products for which the farming practices and meat source in Finland differ most from France (e.g., beef and sheep), we identified products that more closely align with previous datasets made for Finland, e.g., ref. ^51^. Typically, 80% of beef products consumed in Finland are from dairy cattle, resulting in a lower environmental footprint than beef from beef cattle, superficially.

Despite the broader scope of environmental impacts considered by Agribalyse 3.0, we chose to concentrate on a set of indicators (land use, consumptive water use, freshwater eutrophication, marine eutrophication) due to their significance in terms of overall environmental impact and their ability to encapsulate key information about the overall impacts—see also indicators of comparison in ref. ^46^.

Following the verification and updating of product information, LCAs were conducted for each item. The ReCiPe Midpoint (H) method (National Institute for Public Health and the Environment, Netherlands, 2016) provided characterization factors for calculating land use (in square meters of arable crop land equivalents), consumptive water use (in cubic meters), marine eutrophication (in kilograms of nitrogen equivalents), and freshwater eutrophication (in kilograms of phosphorus equivalents).

Marine eutrophication in this context refers to the extent to which emitted nutrients, with nitrogen as the limiting factor in marine waters, reach the marine end compartment. Similarly, freshwater eutrophication refers to the extent to which emitted nutrients, with phosphorus as the limiting factor in freshwater, reach the freshwater end compartment. The assessment of land use in this study focuses on the relative species loss caused by a specific land use type^52^. Consumptive water use represents here the total amount of water consumed, calculated as the difference between the water extracted and the water returned to the environment, across all processes involved in a product’s life cycle.

### Nutrient content of purchases

The retailer’s product groups (*n* = 3435) were linked to nutrient content using the Finnish food composition database v® (version 20, www.fineli.fi), maintained and constantly updated by the Finnish Institute for Health and Welfare. We selected a representative food item from Fineli®, with the assistance of the retailer’s dataset of the most sold items within each food group^53^. The purchase volume (kg) of the product groups was multiplied by the nutrient contents per 1 kg of the food products to obtain the total nutrient contents of the purchased foods.

### Statistical methods

We used sequence analysis to derive protein purchase clusters. For the analysis, we categorized protein sources into the following four groups: i) red meat and processed meat, which also encompassed processed white meat; ii) poultry and poultry dishes; iii) fish and seafood; and iv) plant-based foods, which included plant-protein-rich products and vegetable dishes, excluding whole vegetables^11^. Primary sources at a given time, i.e., the protein source that was the most purchased by the individual in that month, were used as states in the sequence analysis. The analysis identified six clusters with distinguishable purchase preferences for protein sources: Red meat, Red meat mixed (mainly red meat, occasionally poultry and fish), Red meat & poultry, Mixed (all protein sources equally), Fish, and Plant-based^11^.

Expenditure, nutrient content, and environmental impacts per 2500 kcal (10.5 MJ) in each cluster are reported as means and standard deviations (SDs), and characteristics of the population either as numbers and percentages or means and SDs. Distributions within and between clusters of food group-specific expenditure, nutrient content, and environmental impacts are presented graphically and using descriptive statistics (means and proportions). For presentation of the food group-specific results, all the retailer’s 3435 product groups were aggregated into 17 food groups (see Figs. 1–4, Supplementary Data 1,1).

We defined discretionary foods as those that are not essential to meet nutrient requirements, contain a high amount of energy, saturated fat, sugar, salt, or alcohol, and are consumed as snacks rather than a basic part of meals. Because the main aim of our study was related to protein sources, we did not include processed meat in discretionary foods, but showed its role separately. Discretionary foods included sweet bakery products, desserts, savory snacks (including crisps, salted peanuts, etc.), sweets, sweeteners, non-alcoholic beverages (excluding milk-based beverages), alcoholic beverages, cocoa, coffee, and tea. Coffee and tea were classified as discretionary foods because they are non-essential for nutrition and therefore represent an avoidable environmental burden^54^.

In a large sample, such as the current loyalty card data, even small differences become statistically significant. We argue that interpreting the sizes of the differences is more meaningful than their statistical significance. Therefore, we refrain from showing statistical tests when examining differences in food purchases among the clusters. We used the statistical software R (R Foundation for Statistical Computing, http://www.R-project.org/) for the analyses.

Mean differences between the clusters are denoted as described in Eq.^1^:

1 $$\Delta \left(X,Y\right)=X-Y$$

where X and Y are mean values in any two clusters that are compared with each other. The differences are expressed as absolute or relative to Y.

## Supplementary information

Supplementary material

Supplementary Data1

## Data Availability

Supplementary Data 1 contains volumes, expenditures, environmental impacts, and energy contents of the purchases by food group as units per 2500 kcal and as mass percentages of total purchases. Data can be analyzed in collaboration with the research team on reasonable request.

## Code availability

The program codes and their output files are available from jelena.meinila@helsinki.fi.
