# Supplementary material for: Discretionary foods have notable environmental and expenditure relevance across meat and plant protein preferences
Source: NPJ Sci Food. 2026 Jan 20;10:72. doi: 10.1038/s41538-026-00721-x (PMC12921321; doi:10.1038/s41538-026-00721-x)
Supplement: Supplementary file 1 — Supplementary material [file 41538_2026_721_MOESM1_ESM.pdf]

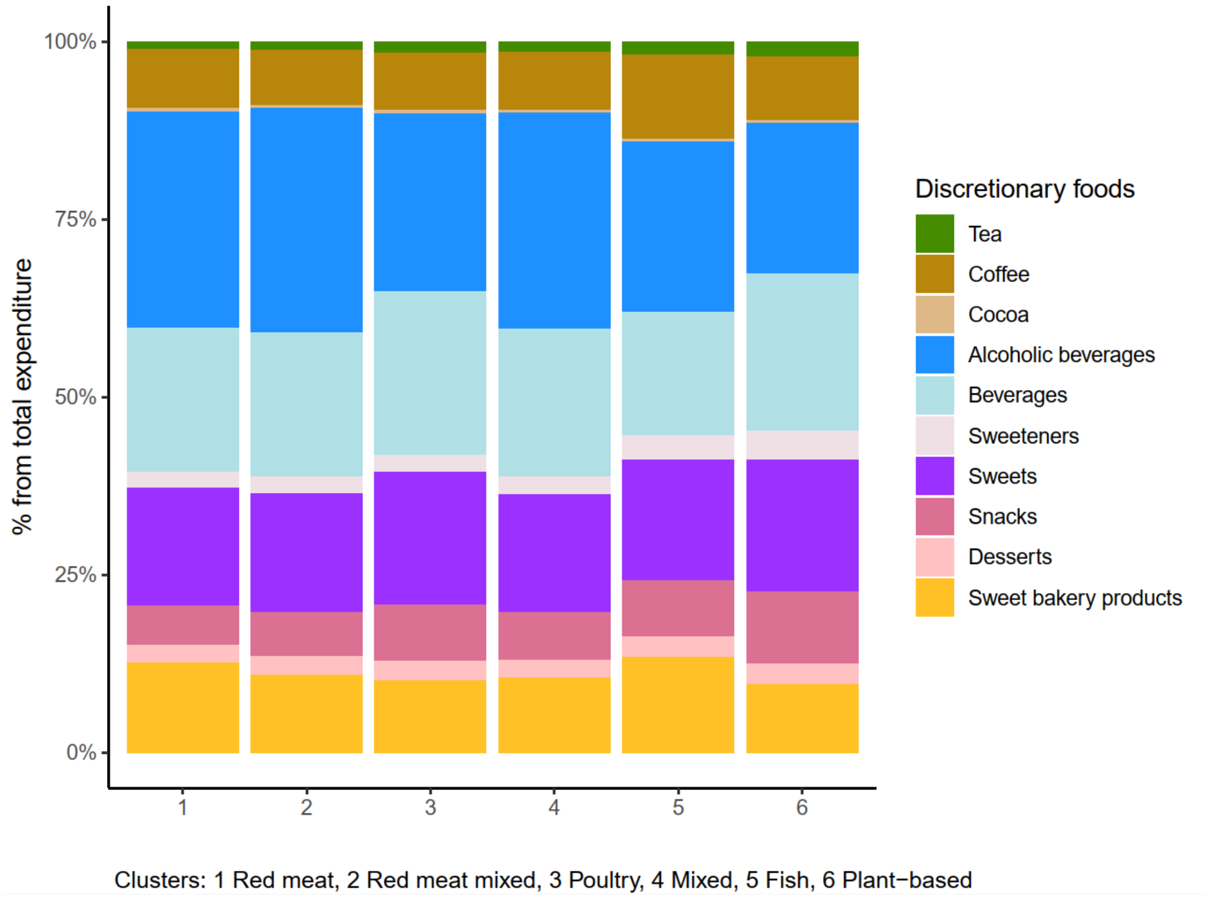

Supplementary Figure 1. Food group specific expenditure (% from total purchases scaled to 2500 kcal) within discretionary foods among 22,901 loyalty-card holders.

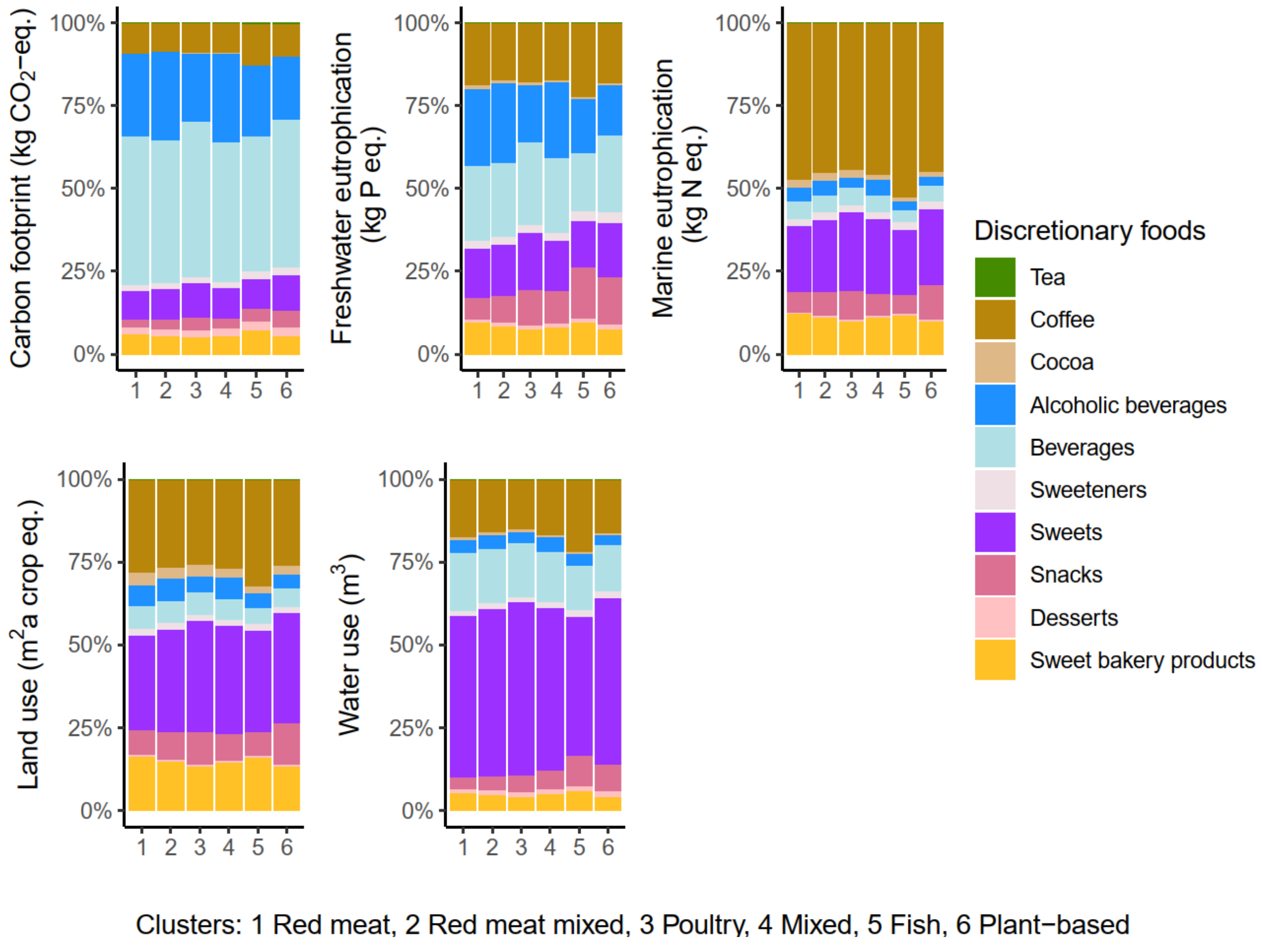

Supplementary Figure 2. Food group specific environmental impacts (% from total purchases scaled to 2500 kcal) within discretionary foods among 22,901 loyalty-card holders.

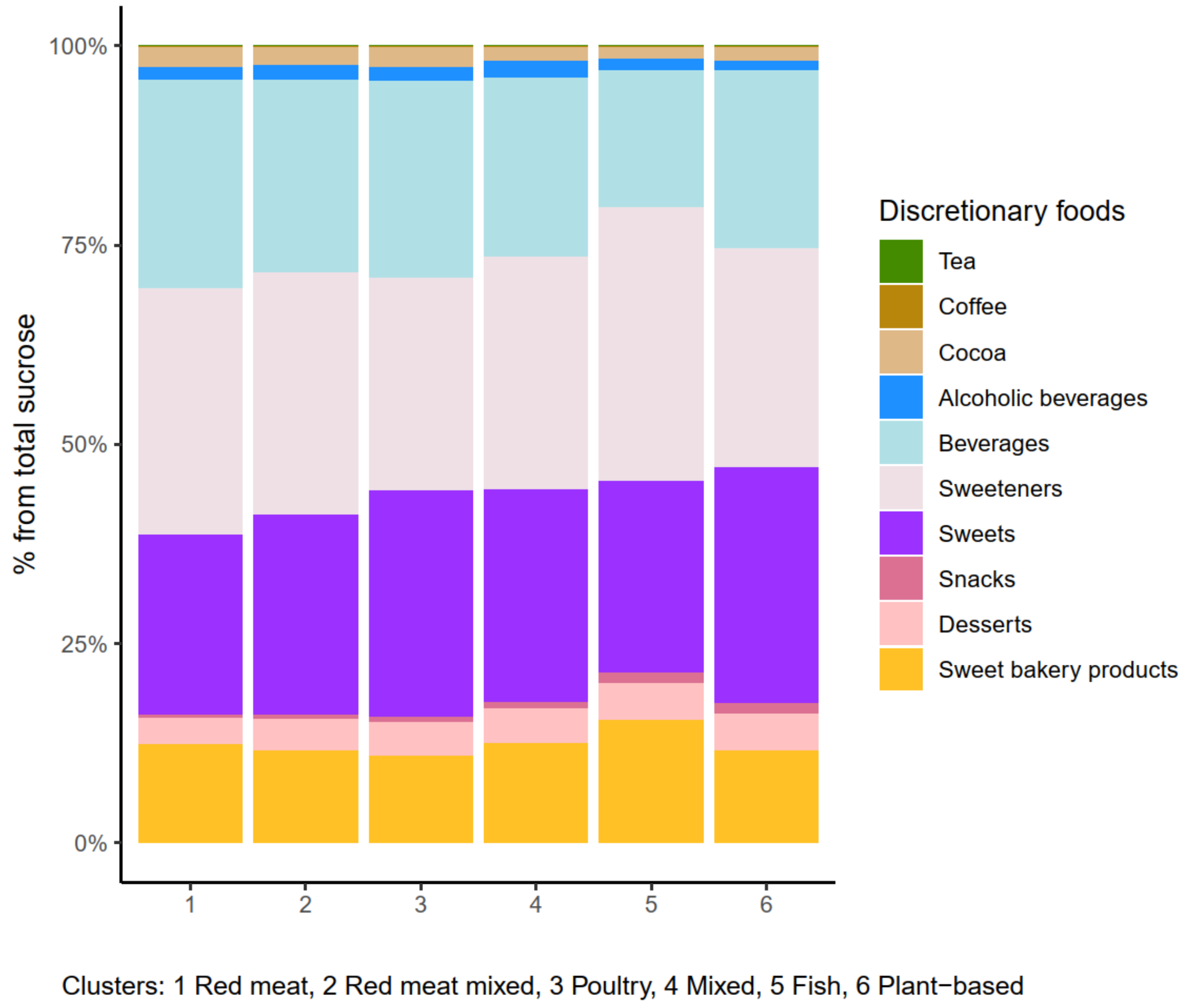

Supplementary Figure 3. Food group specific sucrose content (% from total purchases scaled to 2500 kcal) within discretionary foods among 22,901 loyalty-card holders.

Supplementary table 1. Shares of freshwater eutrophication impact from captured, farmed and uncategorized fish purchases in the clusters.

	Captured fish	Farmed fish	Uncategorized fish*
	% of kg P eq. from total		
Cluster			
Red meat	11	54	34
Red meat mixed	9	54	38
Red meat + poultry	13	53	34
Mixed	2	13	85
Fish	7	55	38
Plant-based	15	49	35

*Fish products for which the species was unknown.
